# The future of multidisciplinary neurotraumatology: Perspectives from the 22^nd^ AMN Congress and the first edition of NTSC Extended - AMN Intensives in Thailand

**DOI:** 10.25122/jml-2025-1004

**Published:** 2025-10

**Authors:** Stefana-Andrada Dobran, Alexandra Gherman, Dafin Fior Muresanu

**Affiliations:** 1 RoNeuro Institute for Neurological Research and Diagnostic, Cluj-Napoca, Romania; 2 Department of Neuroscience, Iuliu Hatieganu University of Medicine and Pharmacy, Cluj-Napoca, Romania

## The Academy for Multidisciplinary Neurotraumatology - advancing research, medical practice, and education

The Academy for Multidisciplinary Neurotraumatology (AMN) is committed to advancing the status of neurotrauma care through its three pillars: research, education, and clinical practice. In 2025, within the new framework of ‘*Think AMN!*’, the Academy introduced two new initiatives to strengthen multidisciplinary collaboration in neurotrauma care and to recognize those who made significant contributions to achieving its mission.

The AMN Focus Groups (FGs) offer an opportunity for experts across the field of neurotrauma to connect and develop tailored action plans aligned with AMN's 2025 Vision and Mission. The Focus groups are a special construct of the Academy designed to incorporate national and international partnerships, cooperation, or collaborations between AMN and scientific bodies or companies related to neurotraumatology, with multidisciplinarity as the basis of this innovative concept.

One of the main goals of the AMN FOCUS GROUPS will be to promote the latest developments in all specific disciplines within neurotraumatology and to actively commit to expanding the scope of AMN educational events.

As multi-purpose tools, the FGs are country-level initiatives that can eventually expand to a regional level and aim to:

- Connect specialists across the ‘Chain of Recovery’ for neurotrauma patients (e.g., emergency medicine, acute care, rehabilitation) at the level of a specific country to strengthen multidisciplinary collaboration;
- Offer support to develop and accelerate country-level initiatives that aim to improve neurotrauma care, with the prospect of scaling;
- Promote multidisciplinary research, education, clinical practice, and advocacy in the field.

The Fellow of AMN (FAMN) distinction is a title offered to a limited number of members who, through their remarkable achievements in research, education, advocacy, and/or clinical practice, enhance the drive of AMN to meet its purpose of advancing neurotraumatology through its multi-specialized fields and fulfill its mission to develop as a scientific organization. This is an exclusive distinction awarded by the Management Committee to honor exceptional service in neurotraumatology.

## NTSC Extended - AMN Intensives - First Edition, Thailand

The Neurotrauma Treatment Simulation Center (NTSC) project began four years ago in Vienna, Austria, where experts from leading institutions managing neurotrauma patients throughout the ‘Chain of Recovery’ developed a multidisciplinary intensive training program. This offered participants the opportunity to visit leading healthcare institutions for one week, experience hands-on simulation exercises, learn from globally-recognized experts, build valuable networks, and ultimately develop treatment plans to improve neurotrauma care standards in their home countries.

**Figure 1:**
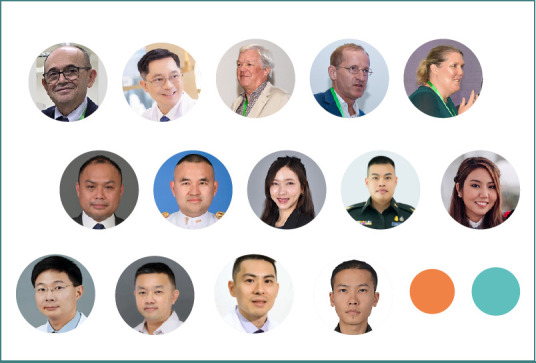
The NTSC Extended - AMN Intensives Thailand Edition faculty: top row - Prof. Dafin Muresanu (Romania), Gen. Dr. Siraruj Sakoolnamarka (Thailand), Prof. Christian Matula (Austria), Prof. Peter Lackner (Austria), Prof. Stefanie Duchac (Germany); middle row: Lt. Col. Dr. Weerawong Sangphosuk, Lt. Col. Asst. Prof. Panu Boontoterm, Dr. Chutima Plukmonton, Maj. Theethawat Sathirarat, Dr. Prinrasar Mongkolkul, all from Thailand; bottom row - Lt. Gen. Clinical Prof. Dr. Kriangchai Prasongsukarn, Col. Dr. Tanongson Tienthavorn, Lt. Col. Dr. Boonchot Kiangkittiwan, and Lt. Col. Dr. Boonchot Kiangkittiwan, all from Thailand.

After three previous successful editions, NTSC launched a one-day training program called NTSC Extended - AMN Intensives, with its first implementation in Bangkok, Thailand. The Phramongkutklao Hospital hosted this trailblazing event on July 3^rd^, welcoming 39 participants from Vietnam, the Philippines, Thailand, Hong Kong, Turkmenistan, Azerbaijan, and Uzbekistan alongside a distinguished Thai and international faculty (Figure 1, Annex 1 A-E). The program provided a deep dive into the ‘Chain of Recovery’ for neurotrauma patients, alongside networking opportunities, bridging connections, and offering hands-on experience.

Annex 1

“It's truly a great honor for us to host this precious program and I'm proud to be part of it. One that we believe we make a meaningful and lasting impact. From the beginning, my colleagues and I have been working closely to prepare for this event which we see as not just a meeting but a movement to elevate neurotrauma education and care [...] We embrace the core philosophy of the AMN: multidisciplinary collaborations. My team and I have come to appreciate just how crucial it is for professionals from different specialties to work together, sharing knowledge, reviewing each other's practice, building new protocols, and creating structural records for future research. This has transformed our approach from focusing only on short-term treatment to delivering long-term team-based care.”

*- General Dr. Siraruj Sakoolnamarka*,

*Co-Director of NTSC Extended - AMN Intensives in Thailand*

**Figure 2:**
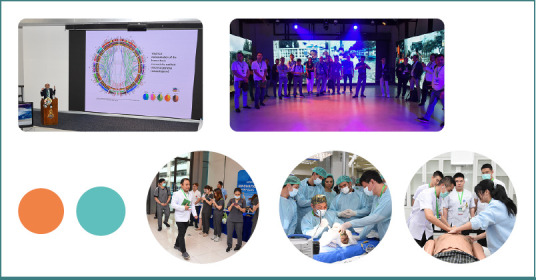
Photos from NTSC Extended - AMN Intensives Thailand edition. First row: Photo from the presentation of Prof. Dafin Muresanu "Multidisciplinary Collaboration in Intensive Care Management for Traumatic Brain Injury, Neuroprotection and Neuroplasticity Strategies" (left), and simulation center (right); Second row: Members of the Thai faculty and participants (left), cadaveric workshop (center), simulation-based workshop (right).

Participants were immersed in key aspects of multidisciplinary collaboration in neurotraumatology. From neuroprotection and neuroplasticity, challenges and advancements in neurotrauma dynamics, intensive care unit management, neuroprotection, and rehabilitation aspects with a focus of dysphagia, presentations focused on covering the entire spectrum of needs of patients with neurotrauma, not only with the consideration of addressing the physical injuries, but reintegrating the patient back into the community and offering a good quality of life (Figure 2). A lunch symposium offered further valuable perspectives on current therapeutic approaches to neuroprotection, and a guided tour of the Phramongkutklao Hospital and Simulation Center allowed participants to better discover the leading neurological trauma center.

“It's a great pleasure to be at the first NTSC – Neurotrauma Simulation Center – in Bangkok. I think it's a big step forward in improving neurotrauma care here in Thailand. It's especially interesting and inspiring to see the people participating and how enthusiastic they are in doing simulations on neurotrauma. So, for me, it's a good opportunity to extend my knowledge about how neurotrauma care is done in Thailand and improve it with my expertise, hopefully.”

*- Prof. Peter Lackner (Austria), program coordinator*

The core part of NTSC Extended - AMN Intensives Thailand edition was represented by simulation scenarios that engaged the participants in hands-on learning through a variety of approaches. Cadaveric workshops helped refine anatomy knowledge and skills and teach life-saving procedures for traumatic brain injury (TBI) patients, while three hands-on simulation stations focused on several vital steps from the management of patients with neurotrauma, including:

- Pre-Hospital and Emergency Aspects;
- Perioperative Aspects;
- Challenges in the Neurological Surgery Intensive Care Unit, and Long-Term Management Aspects (Figure 2).

Participants practiced procedures on realistic mannequins during simulation exercises and learned to better coordinate as teams in high-pressure scenarios (Figure 2).

“I'm happy to say that Bangkok and the Thai people have greatly hosted this event, and I could see that many specialties outside of neurosurgery could learn a lot from this educational event. It highlights the multidisciplinary treatment of neurotrauma, which is actually what we are really after. This simulation course and lectures have shown that it's not just the neurosurgeon who should be involved in neurotrauma, but other specialties as well.”

*- Dr. Lynne Lourdes Lucena (the Philippines), participant*

NTSC Extended - AMN Intensives showcases the importance of multidisciplinary collaboration and practical training throughout the neurotrauma ‘Chain of Recovery’. In the following years, the training program aims to expand to other countries worldwide and implement this approach to handling the neurotrauma patient’s pathway to recovery as standard practice.

## AMN Congress 2025: Advancing multidisciplinary neurotraumatology

**Figure 3:**
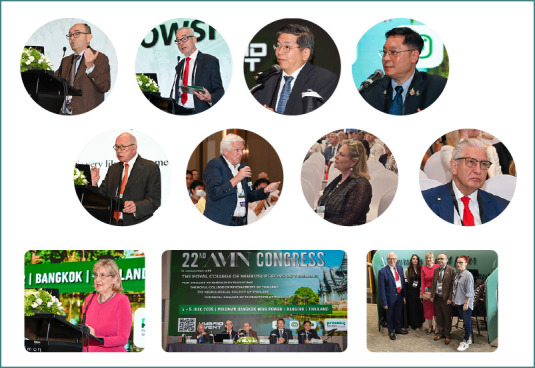
The 22^nd^ AMN Congress (from left to right): top row – Prof. Dafin Muresanu, AMN Secretary General; Prof. Johannes Vester, AMN President; Prof. Kullapat Veerasarn, President of the Royal College of Neurosurgeons of Thailand; Gen. Dr. Siraruj Sakoolnamarka, Consultant Neurosurgeon at Bangkok International Hospital and co-director of NTSC Extended - AMN Intensives in Thailand. Middle row – Prof. Volker Hoemberg, chairman of the AMN Scientific Program Committee; Prof. Christian Matula, Chairman of the AMN Education and Training Committee; Prof. Nicole von Steinbüchel, AMN Past President; Prof. Alexandru Vlad Ciurea, Chairman of the AMN Nomination Committee. Bottom row – Her Excellency Daniela Băzăvan, the Romanian Ambassador to Thailand; the AMN 2025 Presidium; and a group photo of AMN representatives with Her Excellency Daniela Băzăvan.

On July 4-5, in Bangkok, Thailand, following NTSC Extended - AMN Intensives, the AMN Congress brought together, in a hybrid format, over 500 on-site and online participants, including the faculty (Figure 3) comprised of 40 global experts in neurotraumatology, from neurology, anesthesiology, emergency care, rehabilitation, neurosurgery, and many other disciplines. The Congress was organized in collaboration with the Royal College of Neurosurgeons Thailand, the Thai College of Emergency Physicians, the Royal College of Psychiatrists of Thailand, the Neurological Society of Thailand, and the Royal College of Physiatrists of Thailand. With representation from more than 10 countries, including Romania, Azerbaijan, Turkmenistan, Egypt, Hong Kong, South Korea, the Philippines, Poland, Uzbekistan, Vietnam, Thailand, Germany, and Austria, this scientific meeting offered an international platform for sharing the latest research and advancements in neurotraumatology.

The city of Bangkok offered a unique blend of urban life, cultural richness, and scenic beauty, setting the scene for productive discussions and the development of new scientific collaborations.

“The Academy for Multidisciplinary Neurotraumatology is dedicated to open the mindsets to this enlarged approach to communicate the breadth of multiple perspectives simultaneously so necessary to capture the whole picture of neurotrauma and recovery. And that's really new - this way - and it was also extremely appreciated by the participants in this Congress, that's ‘*Think AMN*’.”

*- Prof. Johannes Vester, AMN President*

**Figure 4:**
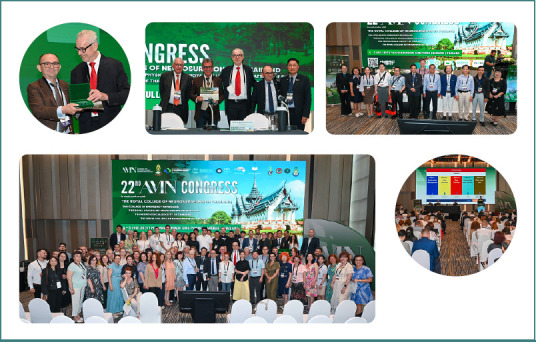
Key moments from the 22^nd^ AMN Congress; (from left to right) top row: Prof. Dafin Muresanu being awarded the status of Fellow of AMN by the AMN President, Prof. Johannes Vester; Prof. Dorel Sandesc received the award for the ‘Highlight of the 22^nd^ AMN Congress’ for his engaging presentations; Group photo with Thai participants and speakers; bottom row: group photo with the participants and speakers from the AMN Congress; photo from the presentations.

**Figure 5:**
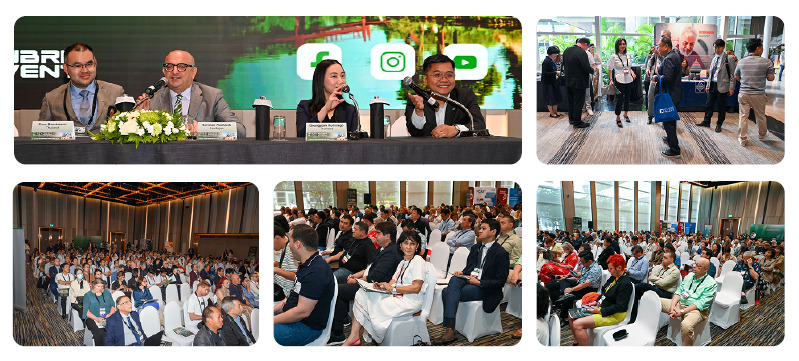
Photos of speakers and participants taken during the 22^nd^ AMN Congress

Based on the principle of multidisciplinarity, the congress (Figure 4, Annex 1 F-I) featured an immersive program that followed the disciplines involved in the patient’s ‘Chain of Recovery’ after neurotrauma. From pre-hospital and intensive care to neurorehabilitation, world-renowned faculty members shared their expertise with the participants throughout the two days (Figure 5). With classic sessions, interactive case discussions, panel discussions, training courses, and a free paper session, the 22^nd^ AMN Congress sought a diversified approach to engage the public and foster a comprehensive understanding of neurotrauma management. All stages of the patient’s recovery pathway were addressed. The program also approached important considerations from healthcare politics, the AMN charter initiative, and the Academy as a global medical society, as well as data and indicators in TBI, and the importance of guidelines through the phases of patient care. A free paper session offered young neuroscientists the chance to present their research, while two training courses addressed essential rating scales in post-neurotrauma cognitive and psychiatric assessment, and provided cross-training specifically for nurses.

“The complexity of neurotraumatology lies at the intersection of precise diagnosis, optimal treatment, and, crucially, rigorous monitoring throughout all stages of recovery to prevent the development of complex neurological affections such as dementia, Parkinson’s disease, or stroke and ensure comprehensive neurorehabilitation. Neurotrauma can diminish the brain reserve, which leads to the need of precise neurological procedures to compensate for the affected cerebral functions and stimulate neuroregeneration, to reestablish its capacities. As the burden of neurotrauma at global level is rapidly rising, strategies are needed to address this public health crisis. In this consideration, the approach of the Academy for Multidisciplinary Neurotraumatology links innovation, latest clinical scientific discoveries, and highly qualified training of human resources that are interconnected within a medical multidisciplinary framework adequate for a high-performance process of diagnosis, treatment, and rehabilitation. Furthermore, it is crucial to create an extensive global framework where communication and interactivity with the world's best neurologists can quickly and consistently supplement any potential inaccuracies that may arise in the treatment of certain complicated medical cases."

*- Professor Dafin Muresanu, AMN Secretary General*

Interviews with distinguished faculty members explored perspectives on the AMN, intakes regarding the congress and NTSC Extended - AMN Intensives, and plans for advancing the AMN vision. These interviews will soon be available online at the AMN Blog and YouTube Channel.

**Figure 6:**
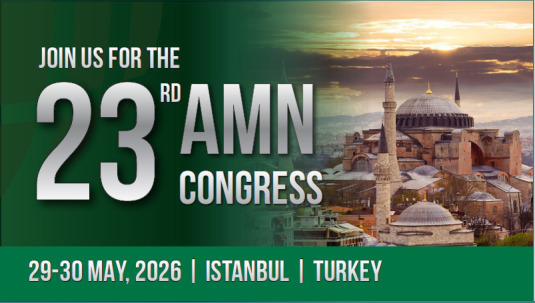
Welcome message to the 23^rd^ AMN Congress in Istanbul, Turkey

In 2026, we welcome you to join the 23^rd^ AMN Congress (Figure 6) on May 29-30 in the picturesque city of Istanbul, Turkey, and help us build the future of neurotraumatology!

## Conclusions

This year, Thailand offered the perfect dynamics for the most important AMN events, the Congress and NTSC Extended - AMN Intensives. In a rapidly shifting environment, the importance of collaboration is ever-increasing, especially in the challenging yet critical field of neurotrauma. Focused efforts should be made in a multidisciplinary framework at local, regional, national, and global levels to improve the status of care, to establish better protocols, increase research, and advocate for patients.

Both NTSC Extended - AMN Intensives and AMN Congress contribute to developing new approaches to neurotrauma, sharing experiences and knowledge, building valuable connections, and creating an optimized ‘Chain of Recovery’ for neurotrauma patients.

*We welcome you to join the AMN and take part in shaping the future of neurotrauma care!*

