# Supplementary material for: The future of multidisciplinary neurotraumatology: Perspectives from the 22nd AMN Congress and the first edition of NTSC Extended - AMN Intensives in Thailand
Source: J Med Life. 2025 Oct;18(10):916–21. doi: 10.25122/jml-2025-1004 (PMC12646196; doi:10.25122/jml-2025-1004)
Supplement: Supplementary file 1 [file JMedLife-18-916-s001.pdf]

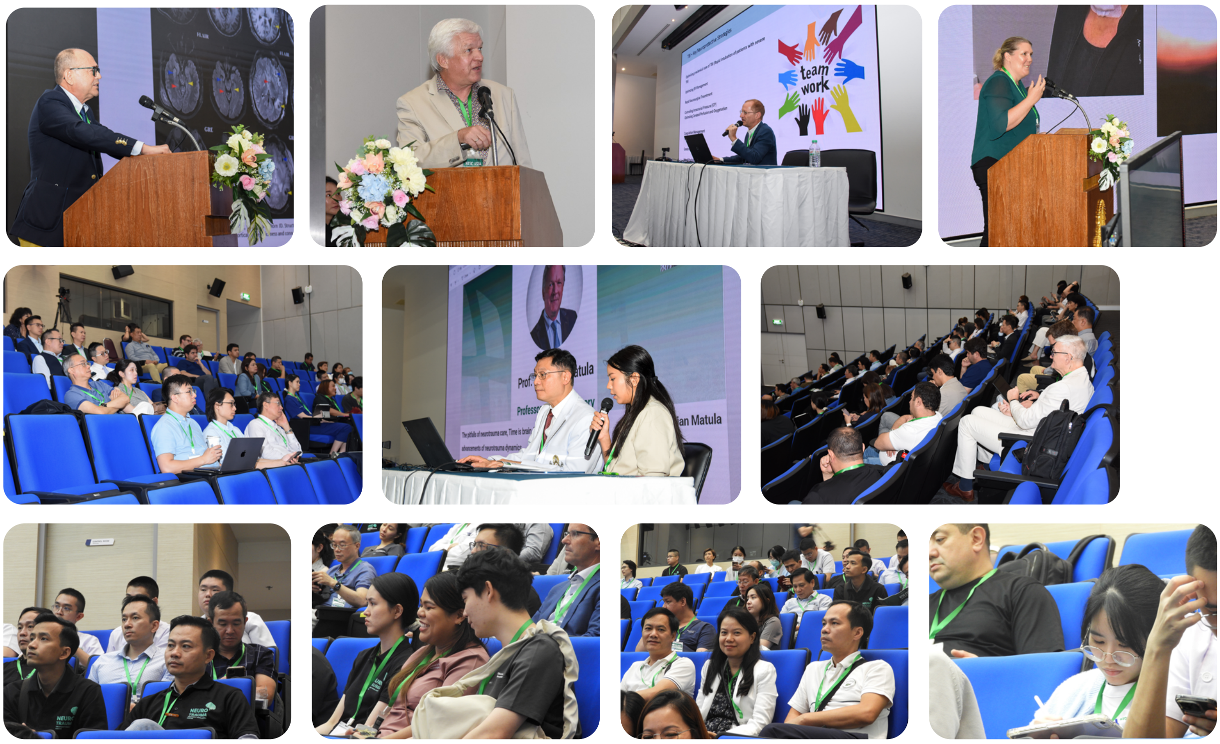


A. NTSC Extended AMN Intensives - First Edition, Thailand. Photos from the presentations and lectures. Top row (from left to right): Prof. Dafin Muresanu, AMN Secretary General and NTSC Extended AMN Intensives Thailand Edition Co-Director; Prof. Christian Matula, Chair of the AMN Education and Training Committee and NTSC Co-Director; Prof. Peter Lackner, NTSC Coordinator; Prof. Stefanie Duchac, NTSC faculty. Second and third rows: photos taken during the event.

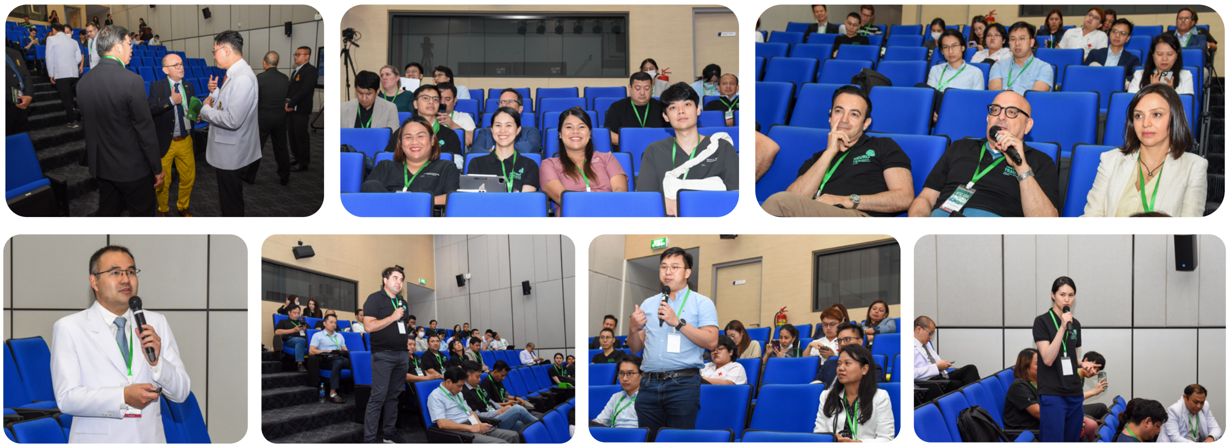

B. Participants and faculty at NTSC Extended AMN Intensives - First edition, Thailand



© 2025 by the authors. This article is an open access article distributed under the terms and conditions of the Creative Commons Attribution (CC BY 4.0) license.

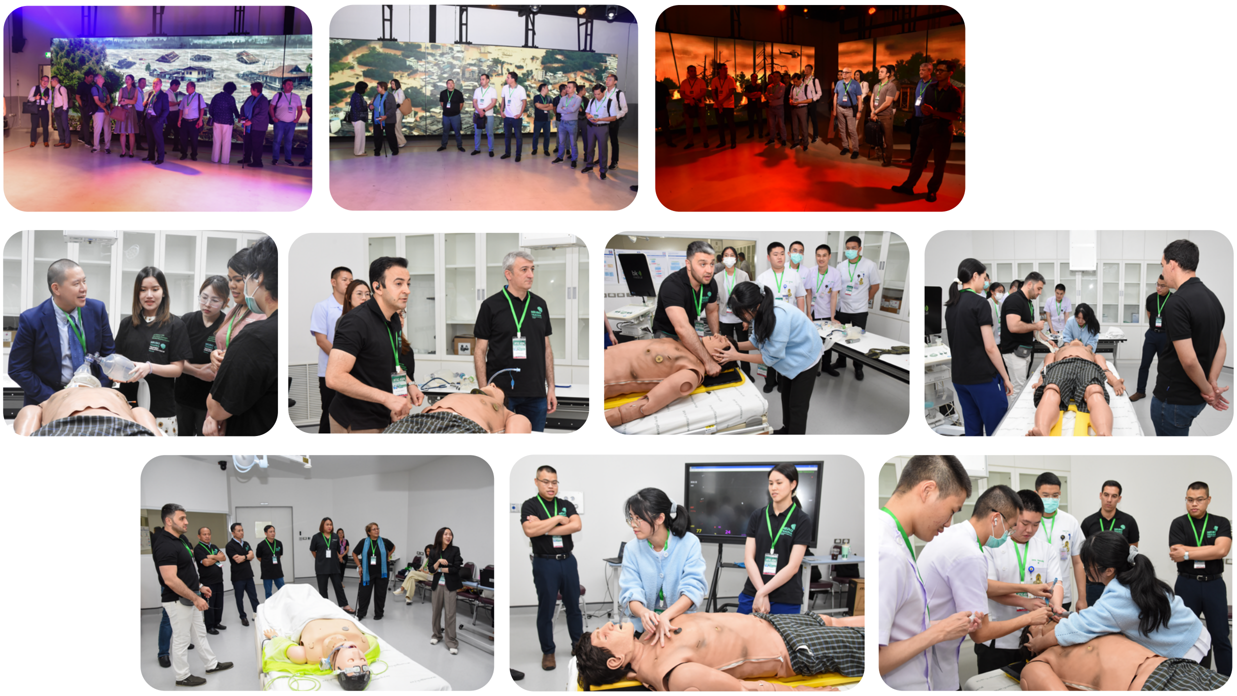

C. The simulation exercises at the NTSC Extended AMN Intensives - First edition, Thailand

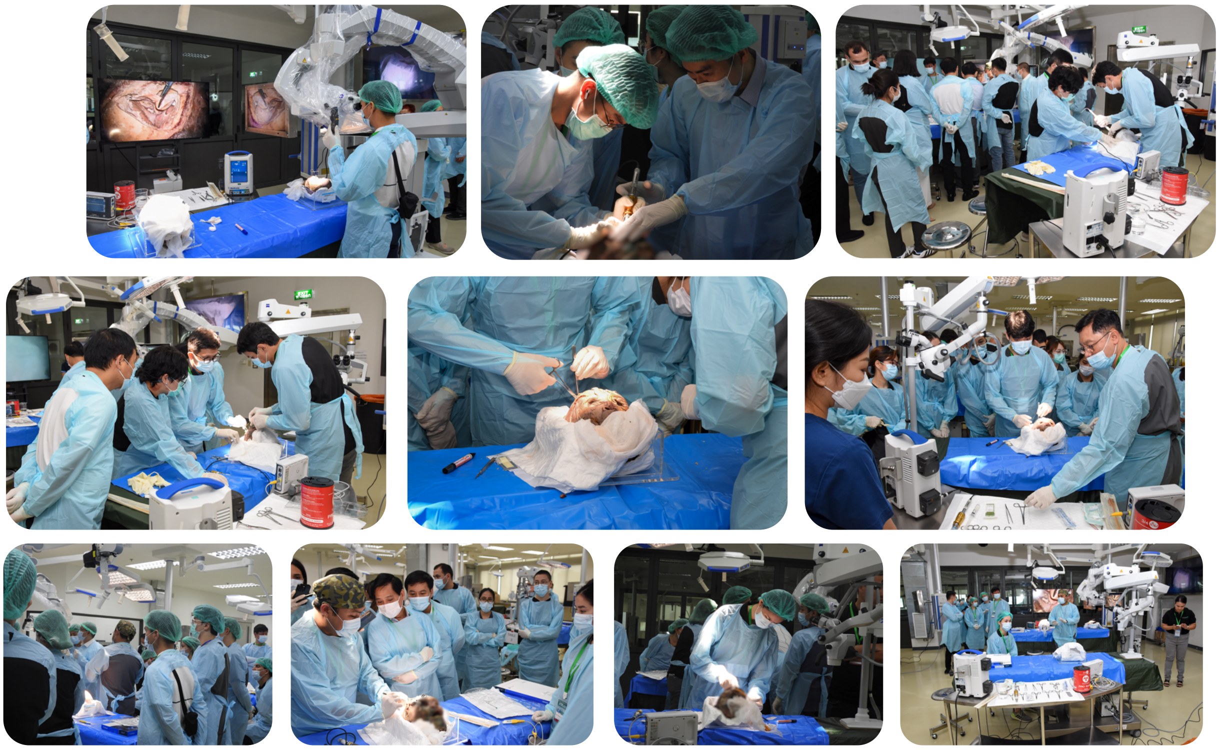

D. The cadaveric workshops at the NTSC Extended AMN Intensives - First edition, Thailand



© 2025 by the authors. This article is an open access article distributed under the terms and conditions of the Creative Commons Attribution (CC BY 4.0) license.

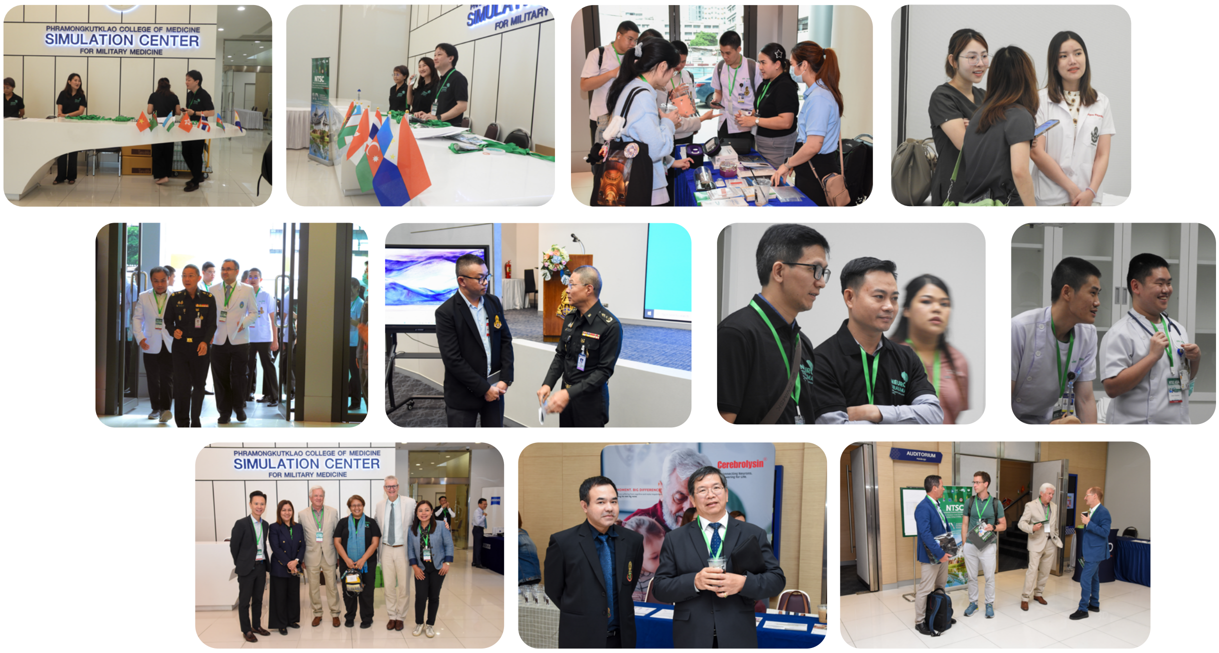


**E. NTSC Extended AMN Intensives - First edition, Thailand - photos of participants and faculty**

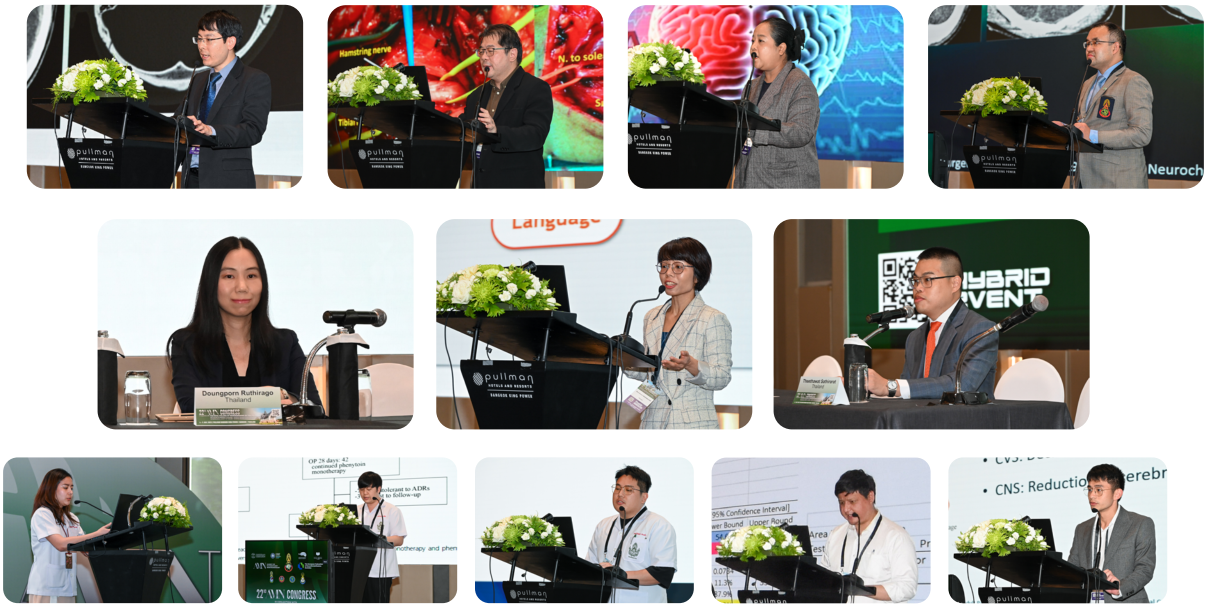


**F. 22nd AMN Congress in Bangkok, Thailand - photos with the Thai faculty. Top row: Dr. Vich Yindeedej, Dr. Bunpot Sitthinamsuwan, Dr. Ann Thaiudom, Lt. Col. Asst. Prof. Panu Boontoterm; middle row: Dr. Doungporn Ruthirago, Dr. Wasineenart Mongkolpun, Dr. Theethawat Sathirarat; bottom row: participants from the Free Paper Session.**



© 2025 by the authors. This article is an open access article distributed under the terms and conditions of the Creative Commons Attribution (CC BY 4.0) license.

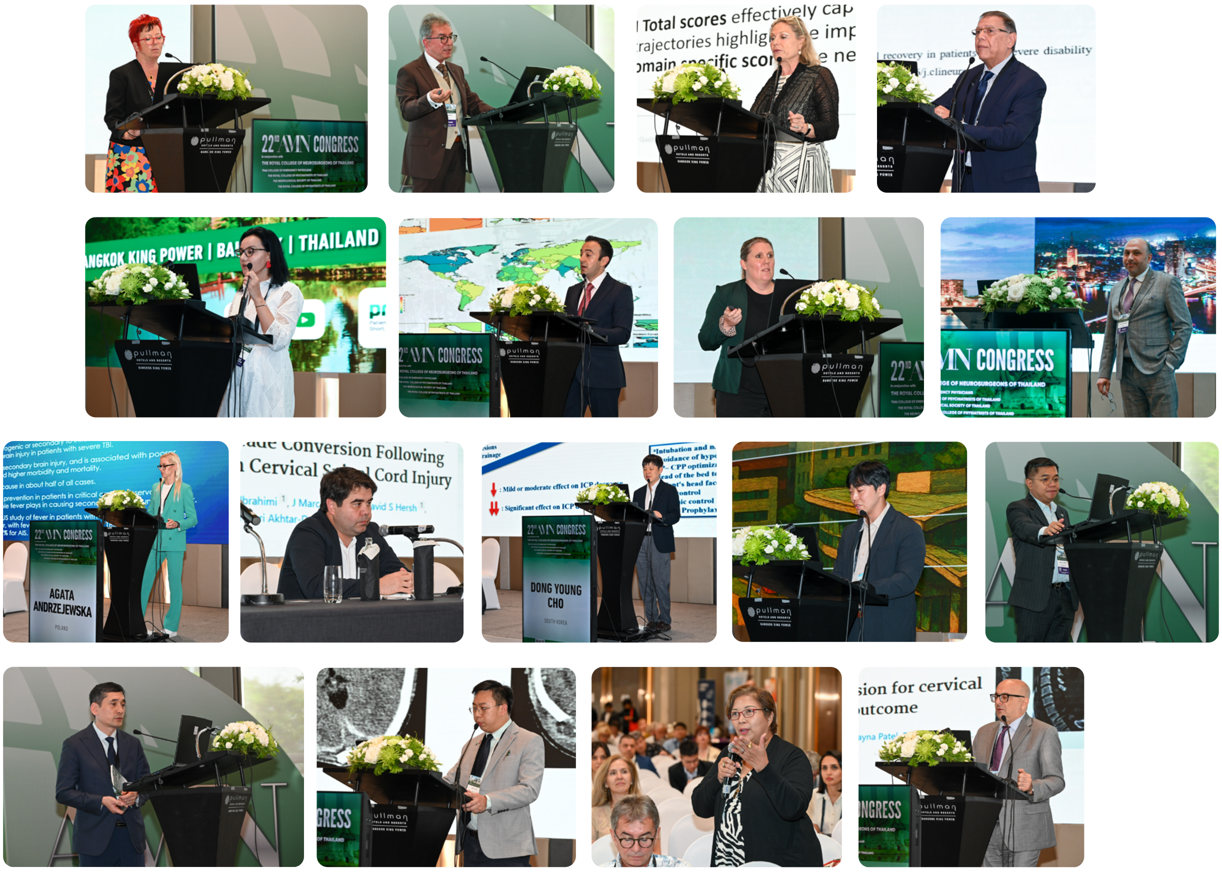


**G. 22nd AMN Congress in Bangkok, Thailand - international speakers. Top row: Dr. Dana Boering (Germany), Prof. Dorel Sandesc (Romania), Prof. Nicole von Steinbüchel (Germany), and Prof. Bassem Boulos Saad (Egypt); middle row; Lecturer Cătălina Crișan (Romania), Dr. Parvin Akbarov (Azerbaijan), Prof. Stefanie Duchac (Germany), Dr. Mohamed Eltantawy (Egypt); second middle row: Dr. Agata Andrzejewska (Poland), Prof. Makhkamjon Makhkamov (Uzbekistan), Prof. Dong Young Cho (South Korea), Dr. Jung Ook Kim (South Korea), Dr. Guillermo V. Liabres (the Philippines); bottom row: Prof. Do Ngoc Son (Vietnam), Dr. Duc Tam Le (Vietnam), Dr. Lynne Lourdes Lucena (the Philippines), Dr. Rovshan Khalizada (Azerbaijan).**

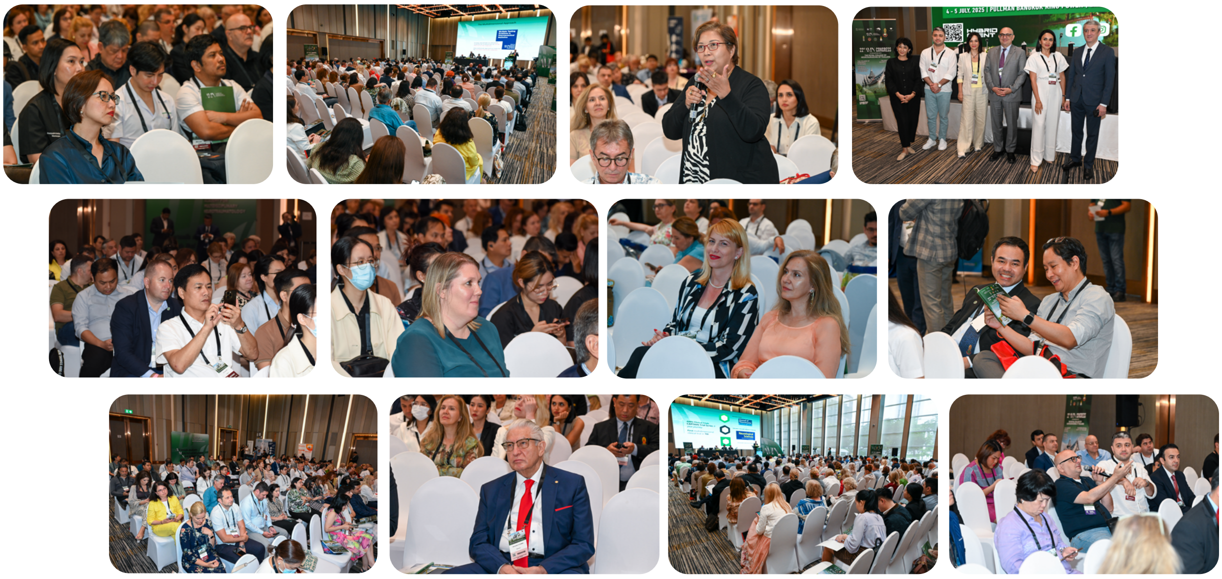

**H. 22nd AMN Congress in Bangkok, Thailand - photos of the participants and speakers**



© 2025 by the authors. This article is an open access article distributed under the terms and conditions of the Creative Commons Attribution (CC BY 4.0) license.

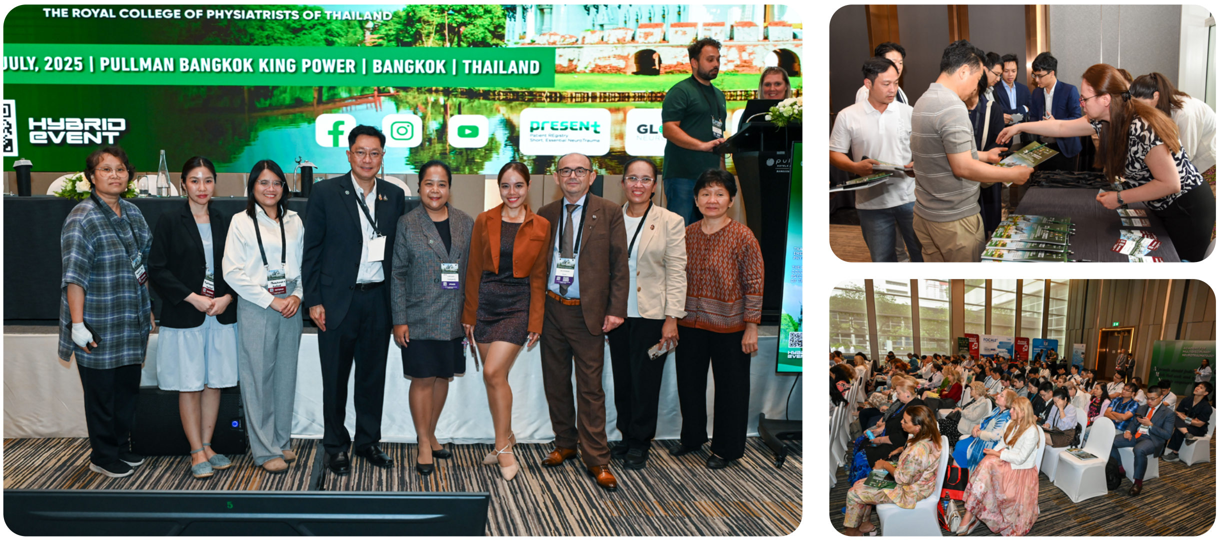


I. The 22nd AMN Congress in Bangkok, Thailand - group photo, photos from the registration and during the congress



© 2025 by the authors. This article is an open access article distributed under the terms and conditions of the Creative Commons Attribution (CC BY 4.0) license.
